# Pyoderma Gangrenosum Simulating Necrotizing Fasciitis

**DOI:** 10.1155/2015/504970

**Published:** 2015-12-10

**Authors:** Erik Friedrich Alex de Souza, Guilherme Almeida Rosa da Silva, Gustavo Randow dos Santos, Heloisa Loureiro de Sá Neves Motta, Pedro Afonso Nogueira Moisés Cardoso, Marcelo Costa Velho Mendes de Azevedo, Karina Lebeis Pires, Rogerio Neves Motta, Walter de Araujo Eyer Silva, Fernando Raphael de Almeida Ferry, Jorge Francisco da Cunha Pinto

**Affiliations:** Federal University of the State of Rio de Janeiro (UNIRIO), Mariz e Barros Street, 775 Tijuca, RJ, Brazil

## Abstract

Pyoderma gangrenosum received this name due to the notion that this disease was related to infections caused by bacteria in the genus* Streptococcus*. In contrast to this initial assumption, today the disease is thought to have an autoimmune origin. Necrotizing fasciitis was first mentioned around the fifth century AD, being referred to as a complication of erysipelas. It is a disease characterized by severe, rapidly progressing soft tissue infection, which causes necrosis of the subcutaneous tissue and the fascia. On the third day of hospitalization after antecubital venipuncture, a 59-year-old woman presented an erythematous and painful pustular lesion that quickly evolved into extensive ulceration circumvented by an erythematous halo and accompanied by toxemia. One of the proposed etiologies was necrotizing fasciitis. The microbiological results were all negative, while the histopathological analysis showed epidermal necrosis and inflammatory infiltrate composed predominantly of dermal neutrophils. Pyoderma gangrenosum was considered as a diagnosis. After 30 days, the patient was discharged with oral prednisone (60 mg/day), and the patient had complete healing of the initial injury in less than two months. This case was an unexpected event in the course of the hospitalization which was diagnosed as pyoderma gangrenosum associated with myelodysplastic syndrome.

## 1. Introduction

Pyoderma gangrenosum (PG) was first described in 1916 by Brocq and better characterized 14 years later by Brunsting [1]. At that time, PG was thought to originate from bacterial infections from the genus* Streptococcus*. Despite this initial assumption and the absence of reliable data about its pathogenesis, today it seems that the etiology of PG is not directly related to any infectious agent [1]. PG cases are considered to be idiopathic in 25 to 50% of the cases [2]. The most currently used definition is that PG is characterized as a rare and often recurring chronic neutrophilic dermatosis [1].

It is estimated that the incidence of PG is between three and ten cases per million people/year [1, 2]. However, PG epidemiology has never been determined by population-based studies and has been formulated only on the basis of case reports and serial cases [3].

Clinically, PG has four main variants: pustular, vegetative, bullous, and ulcerated. The most common form is ulcerated, which begins in the presence of a papule or nodule to an ulcerated lesion and progresses quickly to ulcerated and painful lesions [2]. Such lesions may still be displayed as multiple or solitary with a speckled and/or erythematous appearance [1]. The lower extremities have a higher frequency of involvement [1].

In up to 25% of cases, the appearance of such lesions is associated with prior trauma such as venipuncture, insect bites, injections, and biopsies. These cases can be identified as pathergy phenomena [2].

Despite having no restrictions on age, PG most commonly affects younger adults of the age between 25 and 54 years and affects women more frequently than men. Children are rarely affected (<4% of cases) as their symptoms are normally associated with other systemic diseases [1]. Moreover, there is a predominance of PG emergence in patients with inflammatory bowel disease (Ulcerative colitis (UC) and Crohn's disease), cancer, arthritis, and hematological disorders [1]. Some reports refer to onset in patients who are immunocompromised by medication or have AIDS [3]. PG occurs in approximately 1% to 12% of patients with UC and less commonly in patients with Crohn's disease. The clinical manifestation of PG normally appears after the opening of inflammatory bowel disease; however, there are cases that precede the gastrointestinal manifestations [2].

The main objective in managing PG is to limit tissue destruction, promote wound healing, and obtain a good cosmetic result [1]. A therapeutic approach should include direct and simple cleaning of the injury with subsequent curative antiseptic use, hyperbaric oxygen therapy, topical or intralesional corticosteroids, 6-mercaptopurine or azathioprine, topical cromolyn, dapsone, clofazimine, cyclosporine, sulfa drugs, thalidomide, TNF-*α* inhibitors (infliximab), and systemic corticosteroids, which are considered to be the most effective drugs in the treatment of PG [4, 5]. Specifically regarding the use of corticosteroids, initially high doses of prednisolone (approximately 100 to 200 mg/day) or prednisone (60–80 mg/day) are usually required. Moreover, the surgical management of inflammatory bowel disease may have a role in the treatment of secondary PG in patients who are unstable and refractory to drugs [6].

Thus, because of its wide range of clinical presentations, PG requires intensive multidisciplinary work, often involving dermatologists, plastic surgeons, gastroenterologists, and immunologists, among others [3]. In this context, PG is important in internal medicine as it is a disease with many differential diagnoses. Additionally, there may be disastrous consequences when treatment is delayed, inadequate or insufficient. Mortality associated with PG can reach up to 30% [3].

Necrotizing fasciitis (NF) was first mentioned as a complication of erysipelas by Hippocrates around the fifth century AD. In 1924, the first case was reported by Meleney [7]. The disease is characterized by a severe and rapidly progressive soft tissue infection causing necrosis of subcutaneous tissues and fascia [8–10].

The pathogenesis of NF involves complex interactions between the agent and the host. Although it may also occur in previously healthy individuals, NF is more prevalent in individuals with risk factors for infections, such as diabetics, alcoholics, and intravenous drug users, as well as those with chronic liver disease or renal insufficiency or who are obese, elderly, or immunocompromised [11, 12].

As in PG, NF is usually induced by an injury or local pathological condition, including trauma, wound infection, burns, ulcers, abscesses, lesions caused by parturition, tattoos, insect bites, and acupuncture [10, 11, 13, 14]. However, in some cases NF can start without any preceding trauma or associated pathology [15]. NF is a polymicrobial disease, which can be caused by a number of anaerobic and aerobic facultative bacteria [14, 16]. The synergy between these bacteria may be responsible for the fulminant course of the disease [16].

The diagnosis of NF in its early stages is not always possible and can be confused with simple skin infections such as cellulitis [17]. The most common sites for NF are the abdomen, upper limbs, lower limbs, and perineum [18].

The treatment of NF consists of early diagnosis, radical surgical debridement of all necrotic tissues, broad spectrum parenteral antibiotic therapy, and general measures of aggressive support [19]. Some studies show that early supportive care, such as controlling hypotension and organ dysfunction that result from severe sepsis, and nutritional support and the prevention of thromboembolic events, are as important as the other therapeutic procedures [20].

In the treatment of NF, penicillin is the antibiotic of choice as it is effective for streptococcal infections and has a broad spectrum of action. However, the use of clindamycin may be better [21]. Clindamycin is an antibiotic commonly used to treat severe infections caused by* Streptococcus pyogenes* [20]. The recommended dose of clindamycin ranges from two to four intravenous grams, divided into four doses per day, starting as soon as possible. Penicillin G is recommended at a dose of 12–16 million units per day in four divided doses [20]. In case of suspected infection by anaerobic or mixed bacteria, treatment should be associated with an aminoglycoside or metronidazole. In cases of suspected polymicrobial infection, treatment should include imipenem/cilastatin, ticarcillin/clavulanate, or piperacillin/tazobactam. It is also possible to include medicines that inhibit cytokine production, such as intravenous corticosteroids, gamma globulin, and anti-TNF-*α* antibodies, as well as other therapeutic measures such as hyperbaric oxygen therapy. Currently, amputation is only performed in cases of severe necrosis that are refractory to treatment with irreversible hemodynamic complications [20].

Although rare, NF can cause severe and fulminant disease, requiring early diagnosis and the appropriate therapy [10, 16]. This disease is strongly related to a risk of death (15–50%) and permanent disability through the loss of the affected limb [22].

This study reports the case of a woman who presented with skin lesions whose main differential diagnosis was PG versus NF and reinforces other cases described elsewhere [21, 23–25]. Written informed consent was obtained from the patient for publication of this case report and accompanying images.

## 2. Case Report

A 59-year-old female resident of the Cascadura neighborhood of Rio de Janeiro (RJ/Brazil) was admitted to the 10th ward of Gaffrée e Guinle University Hospital. She arrived with asthenia associated with minimal effort dyspnea, stable angina, blurred vision, nausea, hair loss, and a heavy-leg sensation. She claimed that the symptoms had started a year previously, and she was hospitalized many times because of similar symptoms.

Her medical history included megaloblastic anemia, and she had been treated for refractory cytopenias with multilineage dysplasia (myelodysplastic syndrome) in a hematology clinic. This condition was diagnosed by peripheral blood with cytopenias and less than 1% blasts with no Auer rods and by bone marrow analysis with multilineage dysplasia, ring sideroblasts, less than 5% blasts, and no Auer rods. Additionally, she presented with hypothyroidism for which she received 50 mcg/day of Puran T4, a stable angina that was treated with a statin and nitrates, and a nontreated pulmonary emphysema (37-year smoker). She received a hysterectomy when she was 32 years old because of a uterine fibroid. Additionally, she had a left ileocolectomy 6 months earlier due to the appearance of an inflammatory mass in the hepatic angle of the colon which was found during a colonoscopy.

On admission, she presented with normal vital signs and a regular general status. She was anicteric, acyanotic, eupneic in rest, and discolored (3+/4+) and had a regular-3-time-cardiac rhythm (B3) and a threadlike peripheral pulse.

Hospitalization was requested with complete blood count, hepatic and kidney function tests, a coagulation test, an inflammatory activity test, electrolytes, hemolysis markers, and gasometric analysis. The results are shown in Table 1. At admission, a 2-concentrate-red blood cell transfusion, inhaled corticoids with long-continuance beta agonist, nitrate, statin, and aspirin were prescribed.

The patient improved progressively from her initial condition. However, in the third day of hospitalization she presented a pustulous, erythematosus, and painful injury in her right cubital venous puncture, which evolved into an erythema involved sore.

She developed fever, tachycardia, and tachypnea, and her general status worsened. After the initial symptoms appeared, a broad spectrum antibiotic was given (intravenous-1 g vancomycin every 12 hours and intravenous-4.5 g piperacillin-tazobactam every 6 hours) with the purpose of resolving the bacterial infection. However, there was no satisfactory response.

**Figure 1:**
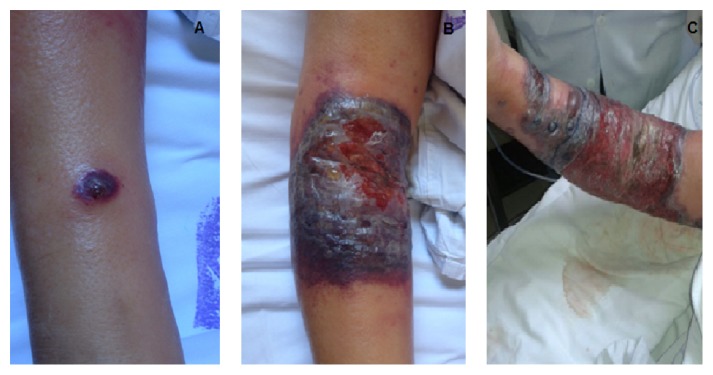
Evolution of lesions in the right antecubital region. (A) A pustular, erythematous, and painful lesion that progressed quickly to ulceration and was circumscribed by an erythematous halo at the site of right antecubital venous puncture. (B) Increased injury refractory to antibiotics. (C) After nine days, the lesion evolved into an erythematous-infiltrated plaque with a hemorrhagic center followed by ulceration circumscribed by erythematous-violet shades of bullous satellite lesions.

**Figure 2:**
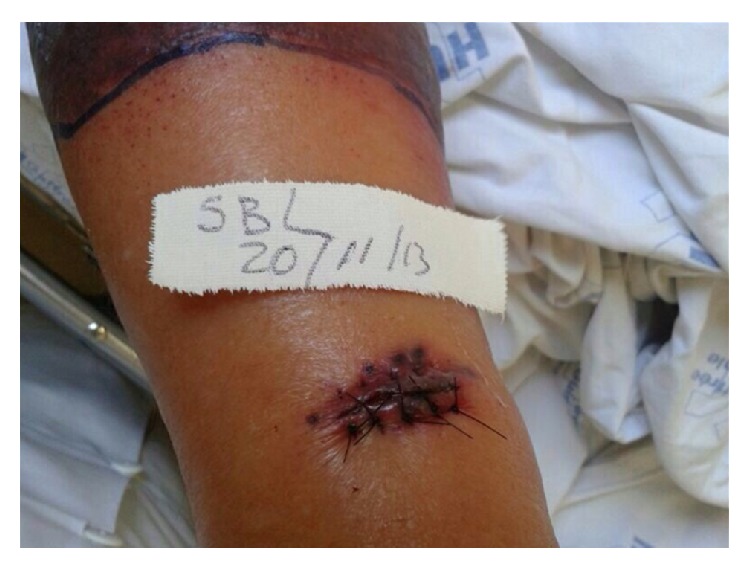
Lesion at the skin biopsy site. Aggravation of the injury at the skin biopsy edges, particularly at the suture sites.

**Figure 3:**
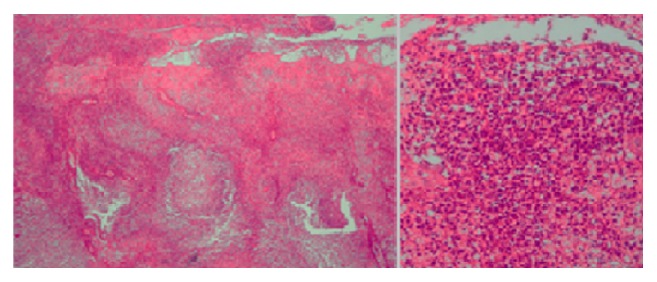
Histopathological injury evaluation by hematoxylin-eosin stain. Necrosis of the epidermis and predominantly neutrophilic inflammatory infiltrate in the dermis.

After approximately nine days, the injury developed into an infiltrated erythematosus plaque with a hemorrhagic nucleus. It then turned into an erythematosus-violet sore with peripheral vesicles. The injury progression is demonstrated in Figure 1. The lesional material was collected for bacterial, mycobacterial, and fungal stains and for culturing and histopathological analysis. The microbiological results were all negative. The histopathological analysis revealed necrosis of the epidermis and predominantly neutrophilic inflammatory infiltration in the dermis (Figure 2). The lesion worsened at the biopsy site (Figure 3).

**Figure 4:**
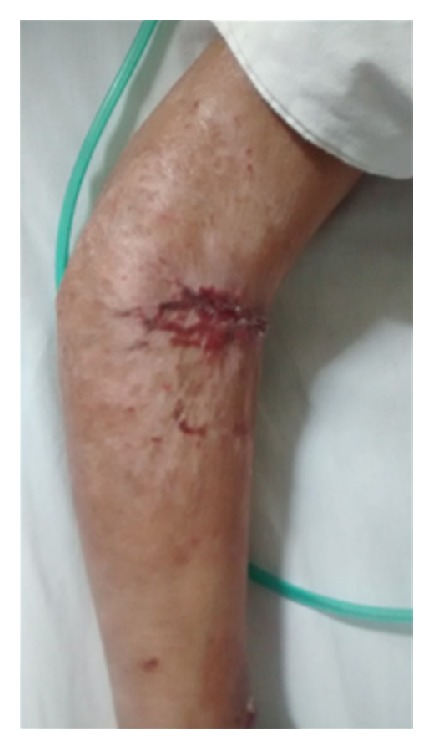
Scar lesion. Appearance of lesion recovery after 45 days of corticosteroid use.

Once PG was suggested as a possible diagnosis, corticoid therapy was started (intravenous-300 mg/day methylprednisolone for three days and then 80 mg/day oral prednisone after the fast improvement). After 30 days, the patient was discharged from the hospital with a prescription for oral prednisone (60 mg/day), which completely resolved the lesion in less than two months (Figure 4).

## 3. Discussion

Despite considerable effort and some scientific advances in the search to better understand the pathogenesis of PG, its underlying cause remains unknown. Nevertheless, given the current evidence, many authors have suggested that PG is an autoimmune disease [1].

As neutrophils are the predominant cells found in PG lesions, PG has been characterized as a type of neutrophilic dermatosis. Moreover, defects in neutrophil functions, such as irregularities in chemotaxis and hyperreactivity, have been reported [26]. Autoantibodies against skin antigens have also been identified; however, it has not been possible to establish a causal relationship between these antibodies and PG. Additionally, several cellular immune responses are inhibited by a serum thermostable factor and are not dialysable in the serum of a patient with PG [26]. Such data converge on the pathogenesis of PG as having an autoimmune mechanism.

In terms of the clinical manifestations of PG, the lesions are essentially characterized by multiple or solitary painful ulcerations that are rapidly evolving and peppered with an erythematous appearance [27]. The lower limbs are the most common sites of involvement [28]. PG usually starts as a painful lump or a deep lesion and appears as a bleeding surface pustule often after minor skin injuries, which characterizes the pathergy phenomenon. In our case, we had an atypical form of PG that overlaps with Sweet syndrome, and the lesion was in the patient's upper limb. Generally, this nodule and pustule are followed by an ulcerated, irregularly painful, inflammation with high margins and dark-red or purpuric coloration with base necrotic granule dotted with small abscesses [29].

In terms of stages, PG can be found in two presentations. One is characterized by a rapid spread of the lesions with intense pain, systemic toxicity, fever, hemorrhagic blisters, pus, and margins with an inflammatory halo. In contrast, some lesions appear without pain, display massive grains within the ulcer, and develop a crust and hyperkeratosis on the margins that can become large. Some create spontaneous regression sites, while others continue to progress [26]. PG has four distinct clinical and histopathological forms: vegetative, bullous, ulcerative, and pustular. The ulcerative form is the most frequent, and the pustular is the rarest [30].

NF develops within the first 24 hours of injury and is characterized by edema, warmth, erythema, and pain, which rapidly spread proximally and distally from the initial focus. In the next 24 to 48 hours, darkening of the erythema occurs and vesicles and blisters with light yellow content form. On the fourth or fifth day, the lesions became necrotic and rapidly evolve to prostration and confusion [26]. These characteristics demonstrate the clinical similarities of PG and NF.

In relation to the reported case, the patient had an acute and rapidly progressive skin lesion associated with clinical signs of toxemia with the subsequent occurrence of local trauma (peripheral venipuncture), which developed areas of erythema, ulcers, and necrosis with a poor general condition. Given these events, the main differential diagnoses were PG and NF. Empirical antibiotic therapy was initiated, primarily to contain the rapid evolution of NF. Complementary exams were performed, which included stains and cultures for fungi, bacteria, and mycobacteria and a biopsy of the lesion for histopathological analysis.

Systemic corticosteroids were administered after the absence of a response to antibiotics. It was possible to rule out NF and confirm PG due to well-defined clinical criteria: no clinical improvement of the lesion with the use of broad spectrum antibiotics; negative stains for fungi, bacteria, and mycobacteria and a lack of microbial growth in cultures; the presence of necrotic and rapidly progressing lesions with violet, irregular borders; and the association of other systemic diseases. The patient also had refractory cytopenias with multilineage dysplasia (myelodysplastic syndrome), a history of peripheral venous puncture after injury, symptoms compatible with the pathergy phenomenon, and histopathological findings with predominantly neutrophilic inflammatory infiltrate. The rapid injury response to corticosteroid administration confirmed the diagnosis of PG. After diagnosis, the past medical history of an inflammatory bowel mass in the hepatic angle after a colonoscopy biopsy and the appearance of a new lesion at the skin biopsy site were assigned to the pathergy phenomenon.

## 4. Conclusion

The reported case was diagnosed as PG associated with myelodysplastic syndrome. We emphasize the importance of the differential diagnosis of PG and NF in internal medicine. The patient began to be monitored as an outpatient and died from acute myeloid leukemia.

## Figures and Tables

**Table 1:** Laboratory tests.

Parameters	06/11/13	Parameters	06/11/13
Red blood cells	**1.52 M/uL**	Amylase	48 (U/L)
Hemoglobin	**4.99 g/dL **	Lipase	18 (U/L)
Hematocrit	**14.8% **	Total bilirubin	1.28 mg/dL
MCV	97.1 fL	Direct bilirubin	**0.33 mg/dL **
MCH	32.9 (pg)	Indirect bilirubin	**0.94 mg/dL **
MCHC	33.8 (g/dL)	ALT	6 IU/L
RDW	**19.9% **	AST	11 IU/L
White blood cells	**3.39 K/uL**	GGT	14 IU/L
Neutrophils	2.01 (K/uL) (59.3%)	Alkaline phosphatase	162 IU/L
Lymphocytes	1.16 (K/uL) (34.3%)	LDH	**952 IU/L **
Monocytes	0.164 (K/uL) (4.83%)	Calcium	8.3 mg/dL
Eosinophils	**0.002 K/uL**	Phosphate	4.8 mg/dL
Basophils	0.054 K/uL (1.6%)	Total protein	6.5 g/dL
Platelets	**118 K/uL **	Albumin	3.8 g/dL
MPV	9.03 (fL)	Globulin	2.8 g/dL
PCT	0.107 (%)	Potassium	4.64 mEq/L
PDW	22.1	Sodium	136 mEq/L
Haptoglobin	109 mg/dL	Chloride	98 mEq/L
Ferritin	**2000 ng/mL**	Magnesium	1.9 mg/dL
INR	1.157	Urea	33 mg/dL
PAT	12.2 s	Creatinine	0.64 mg/dL
PTT	33.3 s	Glycemia	119 mg/dL
Fibrinogen	**1114 mg/dL **	Direct coombs	Negative
PH	**7.468 **	Sedimentation rate	122 mm
Oxygen saturation	96.9%	pCO2	**28 mmHg **
cLactate	1.0 mmol/L	pO2	93.3 mmHg
cHCO3−	22.1	Base excess	−2.7 mmol/L

MCV: mean corpuscular volume; MCH: mean corpuscular hemoglobin; MCHC: mean corpuscular haemoglobin concentration; RDW: red blood cell distribution width; MPV: mean platelets volume; PCT: Plateletcrits; PDW: platelet distribution width; INR: international normalized ratio; TAP: prothrombin agglutination time; PTT: partial thromboplastin time; ALT: alanine transaminase; AST: alanine transaminase; GGT: gamma-glutamyl transpeptidase; LDH: lactate dehydrogenase.
